# Review of Leishmaniasis Treatment: Can We See the Forest through the Trees?

**DOI:** 10.3390/pharmacy12010030

**Published:** 2024-02-08

**Authors:** Moshe Shmueli, Shalom Ben-Shimol

**Affiliations:** 1 Faculty of Health Sciences, Ben-Gurion University of the Negev, Beer Sheva 8410501, Israel; 2 Pediatric Infectious Disease Unit, Soroka University Medical Center, Beer Sheva 8410115, Israel

**Keywords:** cutaneous leishmaniasis, mucocutaneous leishmaniasis, visceral leishmaniasis, adverse events, treatment

## Abstract

There are three known clinical syndromes of leishmaniasis: cutaneous (CL), mucocutaneous (MCL), and visceral disease (VL). In MCL and VL, treatment must be systemic (either oral or intravenous), while CL treatment options vary and include observation-only localized/topical treatment, oral medications, or parenteral drugs. Leishmaniasis treatment is difficult, with several factors to be considered. First, the efficacy of treatments varies among different species of parasites prevalent in different areas on the globe, with each species having a unique clinical presentation and resistance profile. Furthermore, leishmaniasis is a neglected tropical disease (NTD), resulting in a lack of evidence-based knowledge regarding treatment. Therefore, physicians often rely on case reports or case series studies, in the absence of randomized controlled trials (RCT), to assess treatment efficacy. Second, defining cure, especially in CL and MCL, may be difficult, as death of the parasite can be achieved in most cases, while the aesthetic result (e.g., scars) is hard to predict. This is a result of the biological nature of the disease, often diagnosed late in the course of disease (with possible keloid formation, etc.). Third, physicians must consider treatment ease of use and the safety profile of possible treatments. Thus, topical or oral treatments (for CL) are desirable and promote adherence. Fourth, the cost of the treatment is an important consideration. In this review, we aim to describe the diverse treatment options for different clinical manifestations of leishmaniasis. For each currently available treatment, we will discuss the various considerations mentioned above (efficacy, ease of use, safety, and cost).

## 1. Introduction

Leishmaniasis is a neglected tropical disease (NTD), with a spectrum of clinical syndromes. Visceral leishmaniasis (VL), also called Kala-Azar, expresses the dissemination of the Leishmania parasite throughout the reticuloendothelial system [1]. Mucocutaneous leishmaniasis (MCL) is defined as the involvement of naso-oropharyngeal or laryngeal mucosa and creation of ulcers or lesions [2]. Cutaneous leishmaniasis (CL), the most common clinical syndrome in many affected regions, is characterized by painless and chronic ulcers at infected sites of sand fly bites. Cutaneous leishmaniasis has a variable clinical manifestation, linked to several factors, including the individual host immune response [3]. 

The approach to leishmaniasis diagnosis has undergone profound transformation with the integration of molecular diagnostic tools, such as PCR [1]. In contrast to the traditional diagnostic approach, relying on the microscopic pathological examination of parasites in tissue biopsies from infected lesions, molecular testing provides a conclusive identification of the specific species. This holds considerable significance, as distinct species are associated with the varying clinical manifestations mentioned [4]. The ability to pinpoint the involved species enables healthcare professionals to make targeted treatment decisions [1]. 

Once the specific species causing the disease has been identified, the physician must address different considerations when choosing the most appropriate treatment option. The first consideration and most eminent of all is the treatment efficacy against the species recognized. It is important to recall that the same parasite species might have a unique clinical presentation and resistance profile in different areas across the globe [5]. Due to the lack of evidence-based knowledge regarding treatment, physicians often rely on case reports or case series studies in the absence of randomized controlled trials (RCTs) to assess treatment efficacy [6,7]. Furthermore, the evaluation of drug efficacy is even more challenging because of the biological nature of the disease. Even though death of the parasite can be achieved in most cases, the aesthetic result (e.g., scars) can vary [8,9]. The pathophysiology of CL and MCL involves complicated mechanisms of host–parasite interactions [8,10,11]. The immune response plays a crucial role in disease progression. In CL, an exaggerated Th1 response is associated with healing, whereas a Th2-dominated response may lead to chronicity [9,10]. Similarly, MCL is characterized by an inadequate immune response, allowing for parasite dissemination to mucosal tissues. Moreover, the delayed diagnosis, often associated with leishmaniasis, allows for the formation of complications, such as keloids and disfiguring scars, particularly in CL cases. 

The treatment of leishmaniasis is not without potential side effects. Common side effects of anti-leishmanial drugs include systemic effects (e.g., gastrointestinal disturbances such as nausea, vomiting, and abdominal pain, hepatotoxicity, renal toxicity), as well as skin reactions. Furthermore, given the prolonged treatment courses often required, patients may experience cumulative toxicity. It is crucial for healthcare providers to monitor patients closely during treatment and to balance the benefits of therapy with potential adverse effects.

The ease of use of leishmaniasis treatment is a critical consideration, with topical or oral administration often favoured to enhance patient adherence and facilitate effective management of the disease. While VL and MCL must include systemic treatment by oral or parenteral administration, CL can be treated by other options, such as cream or spray. 

Finally, the cost of treatment constitutes a significant factor for consideration. As previously highlighted, the prevalence of the disease is widespread in third-world countries. While the treatment is efficacious, its affordability poses a challenge for many patients. Global health organizations are actively working to tackle this issue. For instance, the Pan American Health Organization (PAHO) has recently issued new guidelines advocating for a briefer and safer treatment regimen for VL in the Americas, thereby enhancing accessibility for individuals in need [12]. 

In this comprehensive narrative review, our objective is to delineate the array of treatment modalities available for addressing the distinct clinical manifestations of VL, MCL and CL. For each existing therapeutic option, we will thoroughly explore four key considerations, including drug efficacy, potential adverse side effects, ease of administration, and cost of treatment. This review takes a practical approach in detailing the considerations for choosing specific treatment, which hopefully will allow physicians in different settings worldwide a judicious management of simple and complex leishmaniasis cases.

## 2. Visceral Leishmaniasis

Visceral leishmaniasis (VL) is a potentially fatal disease, ranking second and seventh in terms of mortality and loss of disability-adjusted life years among tropical diseases, respectively [13,14]. According to the World Health Organization (WHO), almost 13,000 cases of VL were documented in 2020, with more than 90% of them in only seven endemic countries: Brazil, Ethiopia, India, Kenya, Somalia, South Sudan, and Sudan [15,16]. Visceral leishmaniasis is mainly caused by *Leishmania donovani* and *Leishmania infantum*, the latter also called *Leishmania chagasi* in South America. It is critical to recall that all species mentioned can also cause non-visceral manifestations, CL and MCL included [17,18,19,20]. This fact emphasizes the importance of diagnosis of the specific species involved, allowing the physician to identify cases when visceral disease is possible, and to treat accordingly to achieve prevention. Similarly, VL may rarely be caused by leishmania species that are usually causing CL, like *Leishmania tropica* [21,22,23]. As mentioned above, due to the possible grave outcome of VL, a systemic option is the treatment of choice [24,25]. Correspondingly, this increases physicians’ willingness to cope with relatively severe adverse effects of effective treatment. 

### 2.1. Oral Medications

Miltefosine is an oral treatment for VL, with cure rates of 98% [26]. In a phase III trial, the drug demonstrated an efficacy ranging from 94% to 97% for children after 6 months of follow-up, as determined by the density of parasites in bone marrow and/or splenic aspirates. The effectiveness of miltefosine is comparable to that of other agents, given intravenously (IV), such as amphotericin B and liposomal amphotericin B [27,28]. These remarkable cure rates are unparalleled by other oral drugs, and therefore, make miltefosine an attractive option for VL treatment (Table 1).

Toxic effects associated with miltefosine have been tolerable and reversible, although the therapeutic window appears to be narrow [29]. In a phase III trial, gastrointestinal symptoms like vomiting and diarrhoea were observed. Additionally, some patients experienced reversible hepatotoxicity and nephrotoxicity, as indicated by elevated levels of liver transaminases, urea, and creatinine, which typically normalized by the end of the second week of therapy [27,30]. Therefore, the drug is not approved for the treatment of children younger than 12 years old [1]. Nevertheless, although highly effective, relatively safe, and comfortable for patient use, access to miltefosine remains far from secure for some of those needing it the most, with a cost of hundreds of US dollars per treatment in India, for example [31].

Due to the high cost of miltefosine and prior to its development, other oral medications have been suggested as possible treatment for VL. These included anti-fungal “azoles” agents (e.g., itraconazole), antibiotics (e.g., azithromycin), and other drugs (e.g., allopurinol). Despite the benign side effects profile and relatively cheap cost of the drugs mentioned, these treatments were not proven to have reasonable efficacy in RCTs [32,33,34], thus limiting their widespread use.

### 2.2. IV Treatment 

Liposomal amphotericin B has emerged as a promising intravenous treatment for visceral leishmaniasis. This was driven by the fact that liposomal amphotericin B was found to be as effective as amphotericin B deoxycholate, but much safer. Indeed, efficacy studies have shown notable success, with high cure rates, of liposomal amphotericin B for VL treatment [35]. The side effect profile of the drug is marked by tolerable and reversible toxic effects, including fever, chills, vomiting, and reversible nephrotoxicity [36]. While liposomal amphotericin B may be associated with a higher cost compared to some other treatment options (i.e., amphotericin B deoxycholate), its efficacy and relatively manageable side effects contribute to its favourable consideration. It is important to recall that as administration is parenteral, necessitating hospitalization to administer the drug, it might affect patient adherence. 

Although the mechanism is unclear, pentavalent antimonial compounds have been the mainstay of the treatment of VL, MCL, and CL for approximately half a century [37]. Research from 1998 in Sudan revealed that almost all (98%) of 1593 VL patients treated with sodium stibogluconate (antimony–carbohydrate complex) between 1989 and 1995 responded well to treatment. The side effect profile includes pain and swelling upon injection site (the drug can be given by IV or intramuscular [IM] route). Other adverse events noted are pancreatitis, leukopenia, headache, lethargy, myalgia, arthralgia, diarrhoea, nausea, vomiting, nephrotoxicity, and elevation of liver enzymes. The cost of treatment is relatively cheap, compared with miltefosine and Liposomal amphotericin B [31,36,37].

### 2.3. Clinical Correlation and Integration

**Figure 1:**
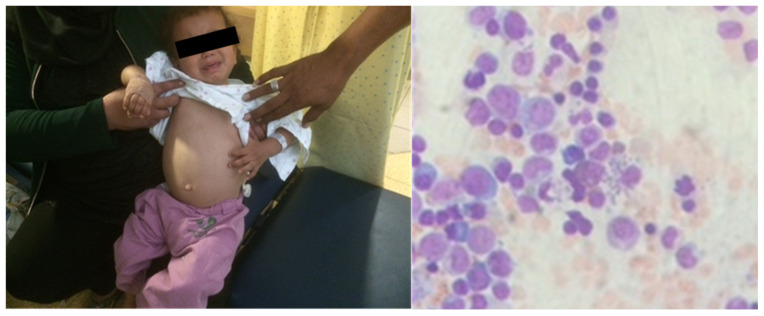
Visceral leishmaniasis in a 4-year-old patient. Hepatosplenomegaly (**left**) and amastigotes in bone marrow biopsy (**right**) are notable.

A 4-year-old patient arrived at our clinic, in a leishmaniasis endemic region, with complaints of prolonged fever (>1 month), abdominal pain and a “swollen belly”. The child was febrile and looked fatigued. A physical examination revealed hepatosplenomegaly. On bone marrow biopsy, characteristic leishmaniasis amastigotes were observed on microscope examination (Figure 1). The PCR result was positive for *Leishmania infantum*. The patient was diagnosed with VL due to an *L. infantum* infection.

The child was treated with IV Liposomal amphotericin B. This therapeutic option is highly effective against this pathogen, as proven in RCTs; in contrast to the favourite oral drug of choice, miltefosine, it is used in young children <12 years of age. With efficacy considerations dominating the decision-making process, we believe that IV Liposomal amphotericin B is the only acceptable option in the case of VL in young children, despite safety concerns (mandating strict monitoring) and the need for hospitalization.

## 3. Mucocutaneous Leishmaniasis

Mucocutaneous leishmaniasis is a chronic inflammatory process involving the nasal, pharyngeal, and laryngeal mucosa, which can lead to extensive tissue destruction [38,39]. Patients should be monitored for associated complications, particularly if the disease progresses. MCL is potentially life-threatening and therefore requires systemic treatment. The disease can develop post-infection from Leishmania species of the Viannia subgenus, typically found in the Americas, including *L. braziliensis*, *L. amazonensis*, *L. panamensis*, and *L. guyanensis* [40,41]. The clinical progression to mucosal disease depends on a combination of host cell-mediated immunity and parasite virulence [42]. Among a population of infected individuals with cutaneous manifestation, infection progresses to the mucosa in 1–10% of patients [43]. The specific host factors that determine which patients will develop MCL are still unclear. Adequate systemic treatment of cutaneous leishmaniasis caused by these species may reduce the risk for mucosal disease, but some risk may remain. The common presenting symptoms include persistent nasal congestion, erythema, and erosions [38,39]. Ulcers can be seen around the nares and lips as the disease progresses. These findings are sometimes mistakenly interpreted for impetigo contagiosum, forcing the physician to be aware of local epidemiology and leishmania species [44].

### 3.1. Oral Treatment

As presented for VL, miltefosine given orally is the treatment of choice. Other treatments elaborated for VL are possible for MCL disease as well (itraconazole, azithromycin, and allopurinol) [1,12,45,46,47]. 

New effective and safe oral drugs are necessary to address the needs of VL patients in developing countries [48].

### 3.2. IV Treatment 

Based on a WHO recommendation, pentavalent antimonial compounds are the most commonly used parenteral medication followed by liposomal amphotericin B [1,12,33,49]. There are no sufficient data about MCL cure rates. Treatment failure and relapse are common [50,51].

Intravenous liposomal amphotericin B cure rates suggest efficacy of 80 to 90% for MCL, similar to rates reported for other antileishmanial drugs (i.e., pentavalent antimonial compounds) [52].

### 3.3. Clinical Correlation and Integration

**Figure 2:**
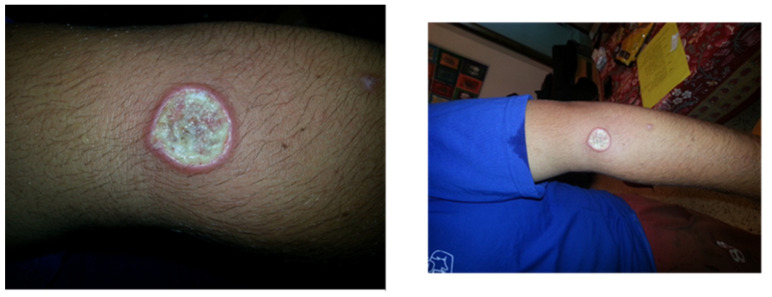
Cutaneous leishmaniasis in a 24-year-old student diagnosed with *L. braziliensis* without adequate systemic treatment; patient is at risk for developing MCL.

A 24-year-old student arrived at our clinic with a recent appearance of an ulcer on his left arm. He just returned from a trip to South and Central America during a summer vacation, including visits to Brazil, Panama, and Guatemala. Physical examination revealed an ulcer, as presented in Figure 2. On biopsy from the lesion, a molecular test was performed, and the PCR result was positive for *Leishmania braziliensis*. The patient was concerned about systemic treatment adverse events, and asked whether topical treatment is sufficient. 

Mucosal leishmaniasis usually becomes clinically evident within several years of the original cutaneous lesions if not treated or treated not optimally [45,53]. Detecting a species, which can potentially cause MCL, should lead the treating physician to try and reduce future complications. Adequate systemic treatment of cutaneous leishmaniasis caused by these species may reduce the risk for the development of mucosal disease [1]. Miltefosine or other systemic drugs found to be effective as discussed above are the treatment of choice.

## 4. Cutaneous Leishmaniasis

Cutaneous leishmaniasis is the most common presentation of leishmaniasis, with an estimated 600,000 to 1 million new patients annually worldwide, with eight countries contributing 90% of CL cases: Afghanistan, Algeria, Brazil, Iran, Pakistan, Peru, Saudi Arabia, and Syria. [54]. Notably, the clinical findings of CL depend, among other factors, on the species involved [15]. Lesions caused by *L. tropica* and *L. major* usually self-heal within a year but tend to leave permanent scars. In contrast, lesions by *L. aethiopica* take years to heal and can develop into severe oral–nasal MCL and diffuse forms of CL [15]. While not posing an immediate threat to life, the recognition and treatment of CL hold significance due to its potential association with the formation of permanent scars. Such scarring can result in aesthetic impairment, diminished quality of life, and enduring psychological consequences, thereby highlighting the importance of addressing this condition [43]. Consequently, the willingness of physicians to administer systemic drugs with relatively severe side effects rely on a combination of often contradicting factors, balancing disease severity and perception by the patient with potential adverse events [15]. 

The complexity of CL underscores the challenges faced by physicians in their attempt to achieve a definitive cure [55]. The mere eradication of the parasite does not necessarily translate into the desired aesthetic result, an aspect subjectively evaluated by the patient. The scarring process itself relies on tangled relationships between diverse factors, such as the time to diagnosis, the unique host immune response, and the parasite-specific virulence [42]. Certain species, such as *L. major*, exclusively induce cutaneous manifestations. Conversely, other species can manifest as CL during the initial stages of MCL [38]. *L. tropica* infection, prevalent in numerous regions, predominantly results in CL, yet it may rarely progress to VL [56]. Consequently, local treatment is usually adequate for CL, but vigilant monitoring is imperative to avert potential complications. 

Various treatment modalities exist for CL. In instances where patient concerns about aesthetic impairment are minimal and there is a low risk of disease progression (e.g., lesions in concealed areas caused by *L. major*), choosing a watchful waiting approach is a valid option. 

Topical treatments are the most common therapeutic options used, but they often bear the possibility of limited efficacy and local adverse events. In selected cases, especially where additional symptoms arise or if the patient has risk factors for disease progression, the physician may opt for systemic treatment [1,6]. Factors associated with choosing systemic treatment may be related to the nature of the skin condition (e.g., multiple lesions, facial lesions, or lesions in hard-to-reach areas, like the eyelid), as well as to patient characteristics (e.g., age, immunosuppression, or prior treatment failure). Finally, physicians may consider systemic treatment when the molecular diagnosis is positive for species associated with VL or MCL [57,58].

The complementary treatment of CL, addressing secondary bacterial infections and residual scarring, is crucial for optimization of the overall therapeutic approach. Antibiotics, both administered locally and systemically, play a significant role in managing bacterial complications that may arise concurrently with leishmaniasis [59]. Given the susceptibility of open lesions to bacterial colonization, especially skin pathogens such as *Staphylococcus aureus*, timely intervention with antibiotics can prevent the exacerbation of the infection, enhance wound healing, and contribute to the overall recovery of the affected individuals [60]. Furthermore, the incorporation of anti-keloid treatment represents another dimension in the complementary strategy for leishmaniasis management [61]. Keloid formation, characterized by the overgrowth of scar tissue beyond the boundaries of the original wound, is a potential consequence of CL. Anti-keloid treatments aim to mitigate excessive scarring, thereby improving the aesthetic outcome for patients.

### 4.1. Local/Topical Treatment

#### 4.1.1. Non-Selective Treatments

Thermotherapy can be a treatment of localized CL. Research conducted in Brazil evaluated its safety and efficacy and found that although most lesions achieved full healing, patients suffered from side effects including itching, burning sensation, pain, and blisters [62] (Table 2).

Cryotherapy, often using liquid nitrogen, is another possible cost-effective and accessible treatment for CL, with variable efficacy. A meta-analysis (2016) comparing clinical trials reveals that the respective per lesion efficacies of 67.3% and 67.7% were reported for cryotherapy and pentavalent antimonial, respectively [63,64]. The most common reported side effects for cryotherapy were hypopigmentation or reversible hyperpigmentation, erythema, oedema, and pain. 

Notably, both thermotherapy and cryotherapy are non-selective treatments. This means that besides killing the parasite, the surrounding tissue cells are also affected and destroyed, causing oedema, erythema and pain, highlighting the need for more directed therapy [63].

Ultraviolet (UV) radiation, a potent suppressor of cell-mediated immune responses, is considered to have some relevance to the treatment of CL. However, the literature on this treatment option is currently anecdotal [65].

#### 4.1.2. Selective Treatments

The WHO recommends using either intra-lesion or systemic pentavalent antimonials (e.g., pentostam) for CL, depending on the specific species and clinical features [12]. In the case of intra-lesional administration, the suggested approach involves injecting 1–3 mL of the medication under and around the lesion until the surface becomes pale, with repeat injections every 5–7 days, totalling 2–5 times. A review from 1999 found that intra-lesional antimonials were successful in partially or completely curing 72–97% of lesions caused by *L. major* [66]. Frequent pain during injections was reported among children [66]. Other reported adverse events include bacterial super-infections, signs of stibio-intolerance in cephalic locations, and progression to more advanced lesions [67]. When choosing treatment by intra-lesional antimonials, the most eminent factor to consider is the procedure’s ease of use. A repeated regimen of injections can be challenging in children, and anaesthesia may be needed for the feasibility of the process, forcing strict monitoring and hospitalization. 

Synthesized nitric oxide-releasing chitosan nanoparticles (NONPs) showed potential in vitro activity against *L. amazonensis* and in infected murine models [68]. Thus, NONPs may be suitable for topical use, and although this path looks promising, further research is needed to achieve precise compounds and administration protocols or devices for clinical use.

Topical paromomycin-containing cream offers a non-systemic treatment for CL. El-On and colleagues developed a formulation with 15% paromomycin in white soft paraffin, also incorporating 12% methyl benzethonium chloride [69]. This formulation proved more effective than no treatment for *L. major* infections in Israel and superior to a vehicle-control treatment for *L. mexicana* and *L. braziliensis* infections in Guatemala [70]. However, due to the high cost and considerable irritancy and intolerance associated with methyl benzethonium chloride (observed in up to 75% of patients), the formulation is infrequently used. A RCT conducted in Tunisia established the efficacy of paromomycin–gentamicin and paromomycin alone for treating ulcerative *L. major* disease [71].

Few reports discuss the use of topical liposomal amphotericin B (LAmB) in treating CL, in contrast to the proven success of intravenous LAmB for VL [72,73,74]. Horev et al. explored this alternative treatment, emphasizing its potential effectiveness, ease of use, and safety. This RCT involved the evaluation of 13 patients with a total of 39 lesions caused by *Leishmania major*, demonstrating LAmB efficacy with only mild and localized side effects [75]. 

### 4.2. Oral Treatment

Systemic treatment for CL is mainly reserved, as discussed above, for complicated cases with previous treatment failure or with specific host and parasite risk factors for treatment failure [1,6]. Briefly, these factors include CL with multiple lesions, facial lesions, lesions in hard-to-reach areas, patients’ young age, immunosuppression and disease caused by leishmania species capable of causing MCL or VL [55,56]. 

Similar to VL, miltefosine given orally is the treatment of choice [12].

### 4.3. IV Treatment

Liposomal amphotericin B has emerged as a promising intravenous treatment for CL when a physician decides that systemic treatment is needed [32,35].

### 4.4. Clinical Correlation and Integration

**Figure 3:**
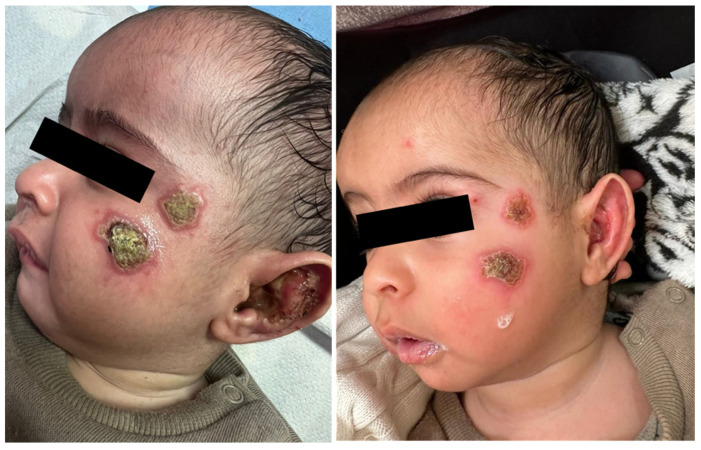
Cutaneous leishmaniasis in a 6-month-old infant, diagnosed with *L. major* infection (**left**). After one week of systemic treatment with IV Liposomal amphotericin B, the patient is showing clinical improvement (**right**).

A 6-month-old infant arrived at our clinic with a recent appearance of multiple facial lesions, as seen in Figure 3 (left). In a biopsy from the lesion, the PCR test was positive for *Leishmania major*. 

This patient presented several factors favouring systemic treatment, including a very young age, multiple facial lesions, and lesions in a hard-to-reach area (the ear auricle). The child was treated with IV Liposomal amphotericin B. This therapeutic option is highly effective against this pathogen, and in contrast to the oral drug of choice, miltefosine, it may be used in children <12 years of age. Figure 3 (right) presents evidence of clinical improvement after one week of treatment.

**Figure 4:**
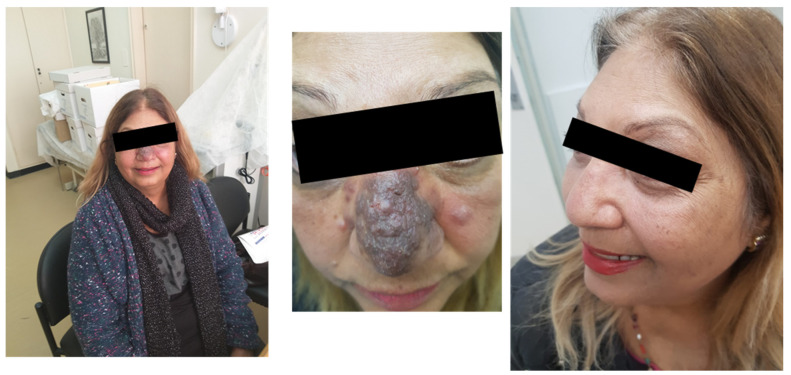
Cutaneous leishmaniasis in a 54-year-old patient (**left**, **middle**). Following miltefosine treatment, the patient showed improvement (**right**).

A 54-year-old patient, diagnosed with *L. major* infection, came to our clinic with a facial lesion on her nose, shown in Figure 4 (left).

Due to the aesthetic nature of the lesion, we decided to use IV Liposomal amphotericin B for treatment. This option is effective, relatively convenient, and generally safe. However, the patient experienced mild side effects, and there was no improvement several weeks following treatment. After consulting the patient, we decided to treat her with miltefosine, an expensive oral medication. Following 28 days of miltefosine treatment, a remarkable improvement was observed, as shown in Figure 4 (right). Notably, changing the treatment might have caused the improvement, but it is also possible that the first drug had some positive (late) effects as well.

## 5. Summary

In this narrative review article on leishmaniasis treatment, the multifaceted landscape of available options is presented. Despite a plethora of choices, a definitive ideal drug for leishmaniasis, and especially for CL, remains elusive. Managing leishmaniasis necessitates a nuanced decision-making process, taking into account various host-dependent and pathogen-dependent factors. Looking forward, there is a significant need for the development of a locally applicable, highly efficacious drug with minimal adverse events. Finally, there is a critical need for more affordable and readily accessible drugs for CL, MCL and VL.

## Figures and Tables

**Table 1:** Systemic treatment (IV and oral) of leishmaniasis.

Drug	Efficacy	Safety (Adverse Events [AE])	Ease of Use	Cost	Other Considerations	Regimen
Miltefosine	+ Proven effect on VL, MCL, CL; not as effective for VL by *L. infantum chagasi*	+ Only for >12 y; GI, nephrotoxicity, hepatoxicity	+ Oral	-	Expensive, 28 days (or more) duration	30–44 kg, 50 mg bid for 28 d; ≥45 kg, 50 mg tid for 28 d
Azoles (fluconazole/ ketoconazole)	- Anecdotal data	+ GI symptoms, headache, hepatotoxicity	+ Oral	+		Adults: 200 mg daily for 6 wk
Allopurinol	- Generally, not effective	+ GI symptoms	+ Oral	+	Usually in combination with another drug	Adults: 300 mg daily for 6 wk
Azithromycin	- Anecdotal data	+ GI symptoms, arrhythmia (rare)	+ Oral	+		Adults: 500 mg daily for 5–10 d
Liposomal amphotericin B	+ Proven effect on VL, MCL, CL	+ Hypotension, nephrotoxicity	- IV	-	Requiring hospitalization	3 mg/kg/day on days 1–5, 14, and 21(total dose 21 mg/kg)
Amphotericin B deoxycholate	+ Proven effect on VL, MCL, CL	- Hypotension, nephrotoxicity	- IV	-	Requiring hospitalization	1 mg/kg per dose daily or every other day for a total of 15–20 doses
Pentavalent antimonial compounds	+ Proven effect on VL, MCL, CL	Pain at injection site (IM), pancreatitis, leucopenia, headache, lethargy, myalgia, arthralgia, diarrhea, nausea, vomiting, nephrotoxicity, and elevation of liver enzymes	- IV/IM	-	Requiring hospitalization	20 mg/kg/day for 21–28 d

Efficacy: + proven efficacy; - generally, not effective/scarce data. Safety: + mild-moderate AE; - severe AE. Ease of use: + easy use; - difficult. Cost: + affordable; - expensive.

**Table 2:** Local treatment of CL.

Drug	Efficacy	Safety (Adverse Events [AE])	Ease of Use	Cost	Other Considerations	Regimen
Thermotherapy	+ Non-specific	- Non-specific, damage to adjacent tissue	+	+		1–3 sessions
Cryotherapy (Liquid nitrogen)	+ Non-specific	- Non-specific, damage to adjacent tissue; residual hypopigmentation	+	+		1–3 sessions
Ultraviolet (UV) light	- Anecdotal data	+ Presumably safe, but scarce data	- Requires specific equipment	-		Not defined
Intra-lesional antimonials (pentostam)	+ Proven effect on different leishmania species	- Painful	-	-	May require anesthesia, hospitalization	Usually 1–5 sessions
Nitric oxide	- Theoretical, scarce data	- Scarce data	- Need for equipment and protocol development	- Unknown		Not defined
Paromomycin-containing creams	+ Proven effect mainly on L. major	+ Pain is common following prolonged duration	+	+	Limited availability	Apply bid for 10 d, rest for 10 d, reapply bid for 10 d
Liposomal amphotericin B (LAmB) ointment	- Limited data	+	+	- Unknown		Not defined

Efficacy: + proven efficacy; - generally, not effective/scarce data. Safety: + mild-moderate AE; - severe AE. Ease of use: + easy use; - difficult. Cost: + affordable; - expensive.

## Data Availability

Data are contained within the article.
